# Evaluating stress resilience of cyanobacteria through flow cytometry and fluorescent viability assessment

**DOI:** 10.1007/s12223-024-01212-w

**Published:** 2024-11-06

**Authors:** Zuzana Kroupová, Eva Slaninová, Kateřina Mrázová, Vladislav Krzyžánek, Kamila Hrubanová, Ines Fritz, Stanislav Obruča

**Affiliations:** 1 Institute of Food Science and Biotechnology, Faculty of Chemistry, Brno University of Technology, Purkynova 118, 612 00 Brno, Czech Republic; 2 Institute of Scientific Instruments of the Czech Academy of SciencesV.V.I., Kralovopolska 147, 612 64 Brno, Czech Republic; 3 Institute of Environmental Biotechnology, Department of Agrobiotechnology, IFA-Tulln, University of Natural Resources and Life Sciences, Konrad-Lorenz-Strasse 20, 3430 Tulln an Der Donau, Austria

**Keywords:** Biotechnology, Cyanobacteria, Flow cytometry, Fluorescent viability probes, Stress resistance, Viability assessment

## Abstract

Cyanobacteria are prokaryotic organisms characterised by their complex structures and a wide range of pigments. With their ability to fix CO_2_, cyanobacteria are interesting for white biotechnology as cell factories to produce various high-value metabolites such as polyhydroxyalkanoates, pigments, or proteins. White biotechnology is the industrial production and processing of chemicals, materials, and energy using microorganisms. It is known that exposing cyanobacteria to low levels of stressors can induce the production of secondary metabolites. Understanding of this phenomenon, known as hormesis, can involve the strategic application of controlled stressors to enhance the production of specific metabolites. Consequently, precise measurement of cyanobacterial viability becomes crucial for process control. However, there is no established reliable and quick viability assay protocol for cyanobacteria since the task is challenging due to strong interferences of autofluorescence signals of intercellular pigments and fluorescent viability probes when flow cytometry is used. We performed the screening of selected fluorescent viability probes used frequently in bacteria viability assays. The results of our investigation demonstrated the efficacy and reliability of three widely utilised types of viability probes for the assessment of the viability of *Synechocystis* strains. The developed technique can be possibly utilised for the evaluation of the importance of polyhydroxyalkanoates for cyanobacterial cultures with respect to selected stressor—repeated freezing and thawing. The results indicated that the presence of polyhydroxyalkanoate granules in cyanobacterial cells could hypothetically contribute to the survival of repeated freezing and thawing.

## Introduction

Cyanobacteria are gram-negative prokaryotic organisms capable of photoautotrophic growth (Cohen and Gurevitz 2006; Juteršek et al. 2017). Historically, cyanobacteria were classified along with algae, so they could be found as ‘blue-green algae’ in older literature. Nowadays, cyanobacteria belong to the phylum *Cyanobacteria* (Cohen and Gurevitz 2006; Koller et al. 2014). Cyanobacteria are ecologically extremely important, as they are capable of oxygenic plant-like photosynthesis (Cohen and Gurevitz 2006; Rasmussen et al. 2008). Cyanobacteria also have a very important function in the ecosystem, as quarter of fixed CO_2_ is being fixed by cyanobacteria (Galloway et al. 2004; Zwirglmaier et al. 2007), and these organisms simultaneously synthesise many interesting secondary metabolites such as glycogen, lipids, various pigments (carotenoids, phycobiliproteins, and chlorophylls) or polyhydroxyalkanoates (PHAs) (Van de Meene et al. 2006). The products of the secondary metabolism of cyanobacteria have the potential to be used as terpene-derived biofuels (Shabestary and Hudson 2016), pharmaceutical substances (Burja et al. 2001) or as packing materials in the food industry (Santos-Merino et al. 2019).

Cyanobacteria cells contain many photosynthetically active pigments such as bacteriochlorophyll *a* and *b*, carotenoids, and phycobiliproteins (Peschek et al. 1989; Nelson and Ben-Shem 2004). The presence of such a broad range of pigments is of advantage for the cyanobacteria but, on the other hand, it also complicates most spectrophotometry-based approaches in cyanobacterial research. For example, the viability assessment of cyanobacterial cells using flow cytometry is complicated by rich autofluorescence signals associated with rich intracellular pigment composition. Strong autofluorescence signal can cover the fluorescent signal of the probe. Nevertheless, autofluorescence can also be beneficial and used for measurement of characteristics of microbial cells as was demonstrated in *Mycobacterium tuberculosis* (Patiño et al. 2008), *E. coli* (Mihalcescu et al. 2015), or to study senescence in *Caenorhabditis elegans* (Pincus et al. 2016).

Cyanobacteria are not typical representatives of prokaryotic organisms as they are capable of oxygenic photosynthesis while their morphology is more complex than that of a typical prokaryotic cell. There are intracellular compartments sometimes called ‘bacterial microcompartments’, which are covered by a layer of proteins in contrast with phospholipid membrane in eukaryotic cells (Nienaber and Steinitz-Kannan 2018). Also, there are another 3 layers of membranes on the surface of cyanobacterial cells to protect the inner intracellular plasmatic system called thylakoid or thylakoid membrane (Vothknecht and Westhoff 2001; Mills et al. 2020; Sen 2020). Cyanobacteria of the genus *Synechocystis* possess an extra surface layer (S-layer) made of proteins with a paracrystalline structure that occurs above the outer membrane (Trautner and Vermaas 2013). Moreover, all cyanobacteria are capable of synthesising glycogen, and many cyanobacteria can also synthesise polyhydroxyalkanoates (PHAs) as the two main carbon storage compounds (Van de Meene et al. 2006). PHAs occur in bacterial as well as cyanobacterial cells in the form of granules (Obruca et al. 2020).

Flow cytometry, the single-cell method, is an analytical technique that became available for research around 1970, and the first algal cultures were investigated in 1978 (Cunningham 1993). In 1984, flow cytometry together with cell sorting was brought to marine biology during symposium organised by European Society for Comparative Physiology and Biochemistry (Holm-Hansen et al. 2013). Flow cytometry has begun to be used as well in aquatic sciences (Yentsch and Horan 1989), and in the same year, flow cytometry has been used for analyses of phytoplankton (Phinney and Cucci 1989). The basic parameters that are measured by flow cytometry are light scattering and fluorescence of each individual cell, which enables counting of the cells and also determination of the viability of the cells. There is a broad range of specific fluorescent probes providing revealing various excitation and emission properties and capable of operating on different principles. For example, SYTOX™ Blue Dead Cell Stain (hereafter SYTOX Blue or SYTOX) is a probe staining dead cells as this probe binds to the DNA of the cells with damaged membranes (Manoil and Bouillaguet 2018). Generally, SYTOX Blue is non-permeable to living cells as the stain penetrates to cells with compromised plasma membranes; therefore, dead cells possess signals with higher intensity. Other dye from SYTOX family is SYTOX Green that is also widely used in the cell viability assays. SYTOX Green can diffuse through permeant membranes, the same as SYTOX Blue is SYTOX Green impermeant only to cells with intact membranes (Rasmussen et al. 2016). Another fluorescent probe is propidium iodide (PI) functioning similarly to SYTOX. In the cells, PI attaches to DNA and RNA so the detected signal is possessed by dead cells or cells with reversibly damaged cell membranes. SYTOX Green was used for viability tests of *Synechococcus* 7002 and *Synechocystis* 6803 (Rosenberg et al. 2019). This method was published in 1999 for the first time (Boulos et al. 1999). Permeability dye calcein was used in *Anabaena* to investigate the heterocyst forming in cyanobacteria. Calcein was used to observe its diffusion between the cytoplasms of all the cells in the filament (Mullineaux et al. 2008).

As a primary storage compound, cyanobacteria synthesise glycogen (Cohen and Gurevitz 2006; Koller et al. 2014). Nevertheless, glycogen is not the only storage compound that cyanobacteria can synthesise; several cyanobacterial species synthesise another storage compound called polyhydroxybutyrate (P(3HB)), polyester material belonging to PHAs family (Van de Meene et al. 2006). PHAs represent a versatile group of polymers since various bacteria can incorporate numerous monomers into PHA chain and plays key role in surviving stress conditions in case of heterotrophic bacteria (Obruca et al. 2020), and the amount of PHA in cell dry mass reaches up to 90% (Fiorese et al. 2009). It has been proven that the presence of PHAs help heterotrophic bacteria in surviving stress conditions such as osmotic pressure, high and low temperatures, and heavy metal stress or UV irradiation (Obruca et al. 2020). For example, in the case of heterotrophic bacteria *Cupriavidus necator* H16, PHAs helped to survive stress conditions such as freezing (Obruca et al. 2016), osmotic pressure (Obruca et al. 2017), and UV irradiation (Slaninova et al. 2018). This fact led us to investigate the relationship of PHA and stress survival also in cyanobacteria. For this reason, we chose cyanobacterial cultures with different PHA content—with high and low (or even none) PHA content. Generally, levels of PHA in cyanobacterial cultures are substantially lower than in heterotrophic prokaryotes. The highest amount of PHA produced by wild type strain of genus *Synechocystis* under photoautotrophic conditions with nitrogen limitation was about 4% (Wu et al. 2001). Amount of PHAs in bacterial cells can be positively affected by the intensity of stress factors and can lead to a targeted and controlled application of the selected stressor known as hormesis effect (Calabrese and Blain 2011; Obruca et al. 2021). Furthermore, mixotrophic cultivations, combining lack of nutrients (N and P) along with introduction of organic substrate, e.g. acetate, can boost PHA accumulation up to 29% in cell dry mass in cyanobacteria (Panda et al. 2006). Except *Synechocystis*, members from other genera capable of PHA production are *Synechococcus elongatus* MA19 (Hein et al. 2001; Mendhulkar and Shetye 2017) or *Chlorogloeopsis fritschii* PCC 6912 (Hein et al. 2001).

In cyanobacterial cells, the cell viability assessment using flow cytometry is complicated by rich autofluorescence signals associated with rich intracellular pigment composition. To our best knowledge, there is currently none established clear and rapid viability assessment protocol for the determination of viability of *Synechocystis* cultures employing flow cytometry. Therefore, in this study, we sought for reliable, easy-to-handle, well-accessible, fast-staining probes for the measurement of the viability of cyanobacterial cells. Cyanobacteria *Synechocystis* sp. PCC6803 and *Synechocystis salina* CCALA192 are both capable of PHA synthesis naturally under various cultivating conditions (Van de Meene et al. 2006; Meixner et al. 2022a). We employed the method to investigate the survival rate of cyanobacterial cultures with various PHA content when exposed to environmentally common and relevant stressor—repeated freezing and thawing.

## Materials and methods

### Microorganisms and cultivations

In this study, two *Synechocystis* strains were used. The brackish wild type (WT) cyanobacteria strain *Synechocystis cf. salina Wislouch* (further abbreviated as CCALA 192) and the WT *Synechocystis* sp. PCC6803 (PCC 6803) were obtained from our partner laboratory of BOKU Tulln, which purchased the strain CCALA 192 from the Culture Collection of Autotrophic Organisms (Czech Republic) and PCC 6803 from Pasteur culture collection (France). Both strains are naturally capable of PHA synthesis; synthesis can be promoted by changing the cultivation conditions (Meixner et al. 2022b). Both strains were cultivated in a medium based on BG-11 medium. This medium has adjusted nitrogen and phosphorous concentrations, which are set to allow a biomass growth until starvation of nitrogen initializes PHA accumulation. A typical colour switch from blue-green to olive-green and orange indicates the nitrogen limitation. The composition of the mineral medium (based on BG-11, code M22O) per litre is NaNO_3_ 0.45 g, Fe(NO_3_)_3_.9H_2_O 0.025 g, MgSO_4_.7H_2_O 0.10 g, CaCl_2_.2H_2_O 0.60 g, Na_2_CO_3_ 0.20 g, K_2_HPO_4_ 0.08 g, and trace element solution 1.50 mL. The composition of the trace element solution per litre was H_3_BO_3_ 0.509 g, CuSO_4_.5H_2_O 0.150 g, KI 0.181 g, FeCl_3_.6H_2_O 0.293 g, MnSO_4_.H_2_O 0.296 g, Na_2_MoO_4_.2H_2_O 0.082 g, NiSO_4_.6H_2_O 0.275 g, Co(NO_3_)_2_.6H_2_O 0.100 g, ZnSO_4_.7H_2_O 0.490 g, KAl(SO_4_)_2_.12H_2_O 0.395 g, and KCr(SO_4_)_2_.12H_2_O 0.470 g (Meixner et al. 2022a, b). The cultures were grown in a 250-mL Erlenmeyer flask in 100 mL medium at 24 ± 2 °C under 16-h light and 8-h darkness light period. The light specification: discharge lamp Philips HPI-T Plus 250W/645 E40, 4500 K (neutral white), 20,500 lm.

To obtain cultivations without and with PHA, both strains were cultivated for 21 and 42 days under autotrophic conditions. The aim was achieving cultures with no/low content of PHA (21 days lasting cultivation) and high content of intracellular PHA (42 days lasting cultivation). In our previous experiments focused on PHA accumulation, we observed that PHA accumulation occurs in the latest phases of the cultivation.

### Characterisation of cyanobacterial biomass

The cell dry mass was used to monitor the cell growth and was determined gravimetrically. The cyanobacterial cultures (2 × 10 mL) were harvested and centrifuged (6000 g × 20 min), and the pellets were dried at 70 °C to constant weight. The pigment isolation and subsequent spectrophotometric determination followed the protocol that was reported in Zavrel et al. (2015), where 1 mL of fresh cyanobacterial culture was harvested, centrifuged, and resuspended in cold methanol in conditions with reduced lights. The samples were incubated for 20 min in the fridge, and then, the absorbance was measured; methanol was used as a blank.

Analyses of PHAs were done in the beginning of the experiment and before the stress was applied. PHA concentration was established using gas chromatography with Flame Ionization Detector (GC-FID) as reported previously (Obruca et al. 2014). The only modification was the amount of sample used for analyses. In the case of cyanobacterial samples, 12–20 mg of dry biomass were used for analyses.

### Testing chosen fluorescent probes

*Flow cytometer*: NL-2000, Cytek Biosciences, equipped with two lasers: blue (488 nm) and violet (405 nm).

*Samples*: 4 cyanobacterial cultures were tested and 3 types of samples were made—live cells, dead cells, 50:50 live:dead cells. Live cells were taken directly from the Erlenmeyer flask, whereas dead cells were prepared by boiling 1 mL of cyanobacterial culture for 10 min. Sample 50:50 was prepared as a mixture of live and dead cells in a ratio of 1:1.

*Sample preparation*: 1 mL of cell culture without stress exposure or after stress exposure was collected and gently centrifuged (3 min, max 3000 g), supernatant was removed, and pellet was resuspended in PBS buffer, pH adjusted if necessary to 7.4 (for 1 L of PBS: 8 g NaCl, 0.2 g KCl, 0.24 g KH_2_PO_4_,1.44 g Na_2_HPO_4_.2 H_2_O, if necessary, pH was adjusted with KOH or HCl). For subsequent measurements, the samples were diluted by the buffer to a cell density of approximately 10^6^ cells per mL. The samples were stained according to the probe-application advised protocol provided by the distributor.

#### Testing the fluorescent probes

Two flow-cytometry dyes have been tested in this study, see the excitation and emission wavelengths in Table 1.

- *SYTOX™ Blue Dead Cell Stain for flow cytometry* (1 mmol/L solution in DMSO, Thermo Fisher Scientific): protocol from manufacturer was used: 1 μL of the probe was added to 1 mL of diluted sample into 10^6^ of cell number, samples were incubated for 5 min in darkness (max 30 min) at room temperature (RT), the final concentration of dye will be 1 µmol/L.
- *Propidium iodide* (PI, Thermo Fisher Scientific): the final concentration of PI was 4 μg in 1 mL of sample. Samples were also diluted to 10^6^ of cell number and the samples were incubated for 15 min in darkness at RT.

**Table 1:** Excitation and emission wavelengths of tested probes

Probe	Excitation (nm)	Emission (nm)	Channel (filter bandwidths)
SYTOX Blue	444	480	V4 (466–481 nm)
PI	535	617	B6 (605–625 nm)

All samples were measured employing spectral flow cytometer in triplicates. Data combining forward scatter, FSC (cell size), and side scatter, SSC (cell integrity, granularity), are not shown. The signals to determine the cell viability were observed in collecting channels. Fluorescent signal of non-viable cells was collected in channel V4 (473 ± 8 nm) for SYTOX Blue and in channel B6 (615 ± 10 nm) for PI (Cytek® Aurora User’s Guide 2019). Data demonstrating the viability of the cells were gained by using the signal from the SSC detector (side scatter channel) and the collecting channel of each probe.

#### Testing SYTOX Blue on real samples

SYTOX Blue was tested to measure the viability of real samples. PCC6803 − was stressed by slow freezing by putting the tubes into − 20 °C without any artificial cryoprotectant and passed two freeze–thaw cycles at − 20 °C. During the assay, 1 mL of cyanobacterial culture was directly transferred into the Eppendorf tube, put into the freezer, and let completely freeze. Subsequently, the samples were withdrawn from the freezer and let melt arbitrarily at room temperature. Subsequently, sample for measuring the viability was taken, washed in the PBS buffer, diluted, and stained by SYTOX Blue as described above. Controls were prepared equally.

Set-up of the controls:

- Sample control—original culture from which samples for freezing were taken. Serves as a control of how viable the culture is—stained by SYTOX.
- Negative control—dead cells without probe evaluating the autofluorescence of the cells.
- Positive control—dead cells with the probe.

### Electron microscopy

#### TEM

The cyanobacterial cultures were prepared as reported in previous work (Mrazova et al. 2023). The cells were centrifuged for 4 min at 6000 g to form a thick pellet and further processed using cryogenic methods. The pellet was pipetted on 3 mm Au/Cu carrier type A pretreated with 1% solution of soy lecithin in chloroform and covered with the flat side of 3 mm Au/Cu carrier type B. The samples were fixed using high-pressure freezing (EM ICE, Leica Microsystems, Vienna, Austria), followed by freeze substitution (EM AFS2, Leica Microsystems, Vienna, Austria). The used procedure was set, as previously described (Kouřilová et al. 2021), to − 90 °C for 72 h; then, the samples were warmed up to − 20 °C for 24 h and the procedure finished, with the final phase at 4 °C for 18 h. Fixed and dehydrated samples were infiltrated with epoxy resin (Epoxy Embedding Medium kit, Sigma Aldrich, Darmstadt, Germany) and cured for 48 h at 62 °C. Embedded samples were cut into ultrathin sections (~ 75 nm) using a diamond knife (Ultra 45°, DiATOME, Nidau, Switzerland) and ultramicrotome (EM UC7, Leica Microsystems, Vienna, Austria). The sections were then stained using conventional staining agents uranyl acetate and lead citrate and observed using a high-voltage transmission electron microscope Talos F200C (Thermo Fisher Scientific, Waltham, MA, USA), equipped with a Ceta-D Camera, using an electron beam of 200 kV voltage.

#### Cryo-SEM

Cryo-SEM samples were prepared and observed in the same way as reported previously (Novackova et al. 2022). For cryo-SEM, the concentrated pellet of cells was pipetted onto 6-mm aluminium carries type A and covered by a flat side of carrier type B without any cryo-protectant or surface treatment and fixed using high-pressure freezing method (EM ICE, Leica Microsystems, Vienna, Austria). The samples were transferred under liquid nitrogen into a cryo-vacuum preparation chamber (ACE600, Leica microsystems), and subsequent freeze fracturing and sublimation (7 min, − 95 °C) were performed. The samples were transferred under vacuum using a shuttle (EM VCT100, Leica Microsystems) into a scanning electron microscope (SEM) equipped with cryo-stage (Magellan 400/L, FEI, Hillsboro, OR, USA) for observation at − 120 °C using 1–2 keV electron beam without any coating (Novackova et al. 2022).

The electron microscopy (EM) study in this work was based on cryogenic fixation (no commonly used chemical fixative was used, but a cryogenic medium for very fast physical fixation). Cells need to be concentrated in a thick pellet, which is then pipetted onto the carrier, which is provided as a part of the high-pressure freezing machine, and the samples are fixed automatically. High-pressure freezing is one of the best fixation methods for biological samples available up to date and does not require any chemical fixation prior to the vitrification of the sample. The high-pressure freezing method enables us to observe the cellular ultrastructure closest to the native state of the cells since there is no addition of chemical fixatives that could shrink or otherwise alter the ultrastructure.

## Results

### PHA accumulation

All the tested cultures were grown autotrophically, particularly two separate cultures were analysed from each strain, and cultures mostly differ in their PHA content regarding their cultivations time (21 days long cultivation for low PHA content in the culture, 42 days long cultivation for high PHA content in the culture). The polymer content was measured from the dried biomass and detected by gas chromatography (GC-FID); this technique is routinely used for quantification of PHA in bacterial cells (Obruca et al. 2014). The results are presented as the average PHA content in the tested population in Table 2. Samples with ‘ + ’ represent the populations in stationary growth phase under starvation of nitrogen which were recognisable by their typical orange-brown colour (Fig. 7 in the Appendix). Samples marked as ‘ − ’represent the populations of cells with low content of PHAs, younger cultures poor in PHA were blue-green or green-coloured. According to biomass amounts, we can suggest both strains grow at the same speed; the most significant difference is in the PHA content, when CCALA 192 accumulated up to 8 mol.% of PHA in the cell dry mass.

**Table 2:** Amount of PHA in used cultures. Population harvested in stationary growth phase contained higher amounts of PHA

Culture	Biomass (g/L)	PHA (mol/mol)	PHA (g/L)
PCC6803 + *	1.43 ± 0.01	**2.58** ± 0.93	0.028 ± 0.013
PCC6803 − *	0.78 ± 0.10	0.1 ± 0.012	0.0008 ± 0.0005
CCALA192 + *	1.45 ± 0.04	**8.21** ± 0.01	0.119 ± 0.012
CCALA192 − *	0.68 ± 0.02	0.09 ± 0.01	0.0006 ± 0.00045

*Cultures marked with ‘ + ’ represent populations with significantly higher PHA content (bold), cultures marked with ‘ − ’ represent the populations of cells with low PHA content in the mass. Results shown with standard deviation. The highest amounts of PHA (bold) were detected in the cultivations marked as ‘ + ’, which were harvested in the stationary growth phase. Cultivations harvested in early exponential phase were poor in PHA content and marked as ‘ − ’

### Pigment content

**Fig. 1:**
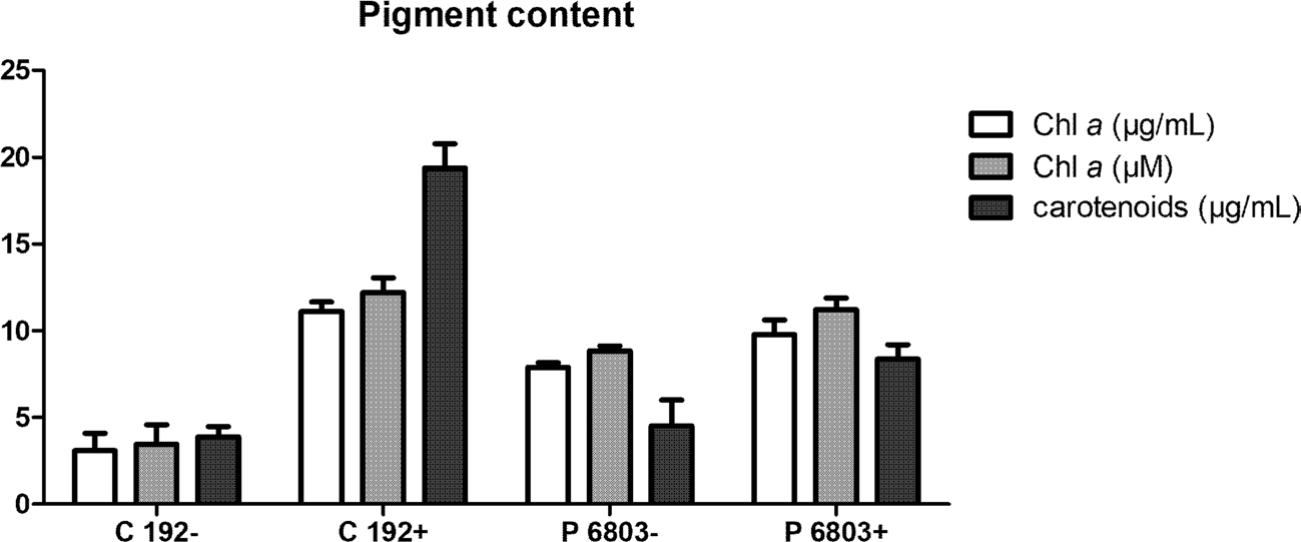
Pigment content of CCALA 192 (C) and PCC 6803 (P) cultures poor (green −) and rich (yellow +) in the content of PHA. The error bar represents the standard deviation. The most substantial difference was observed in case of carotenoids between C 192 − and C192 + . Later stage of cultivation (‘ + ’) led to increase in carotenoids as well as chlorophyll content

In this study, each of the cyanobacterial cultures regarding different ages and amounts of PHAs was analysed for pigment contents such as chlorophyll *a* (Chl *a*) and carotenoids as shown in Fig. 1. Young green cultures with low PHA content (marked as ‘ − ’) contained lower amounts of carotenoids compared to older yellowish cultures with higher amounts of PHA (marked as ‘ + ’). In the case of CCALA192 + (C 192 +), the amount of carotenoids is almost 4 times higher than in CCALA192 − (C 192 −) and twice higher than in PCC6803 + (P 6803 +). Greater differences in the pigment content were observable in strain CCALA 192. We could spot an increase in chlorophyll as well as carotenoids after 3 weeks longer cultivation.

### Identification of suitable fluorescent probe

#### Testing the fluorescent probes

All three selected probes (PI and SYTOX Blue) were tested on the same samples prepared from the above-mentioned cyanobacterial cultures, specifically the samples included two different strains of cyanobacteria, but the cultures also differed in PHA contents due to different cultivation times (see chapter PHA accumulation). The tested cultures were PCC6803 + , PCC6803 − , CCALA192 + , CCALA192 − , each culture in all 3 sample versions—viable, dead, and mixed 50:50. The signal was observed in the corresponding fluorescent channels (Table 1).

**Fig. 2:**
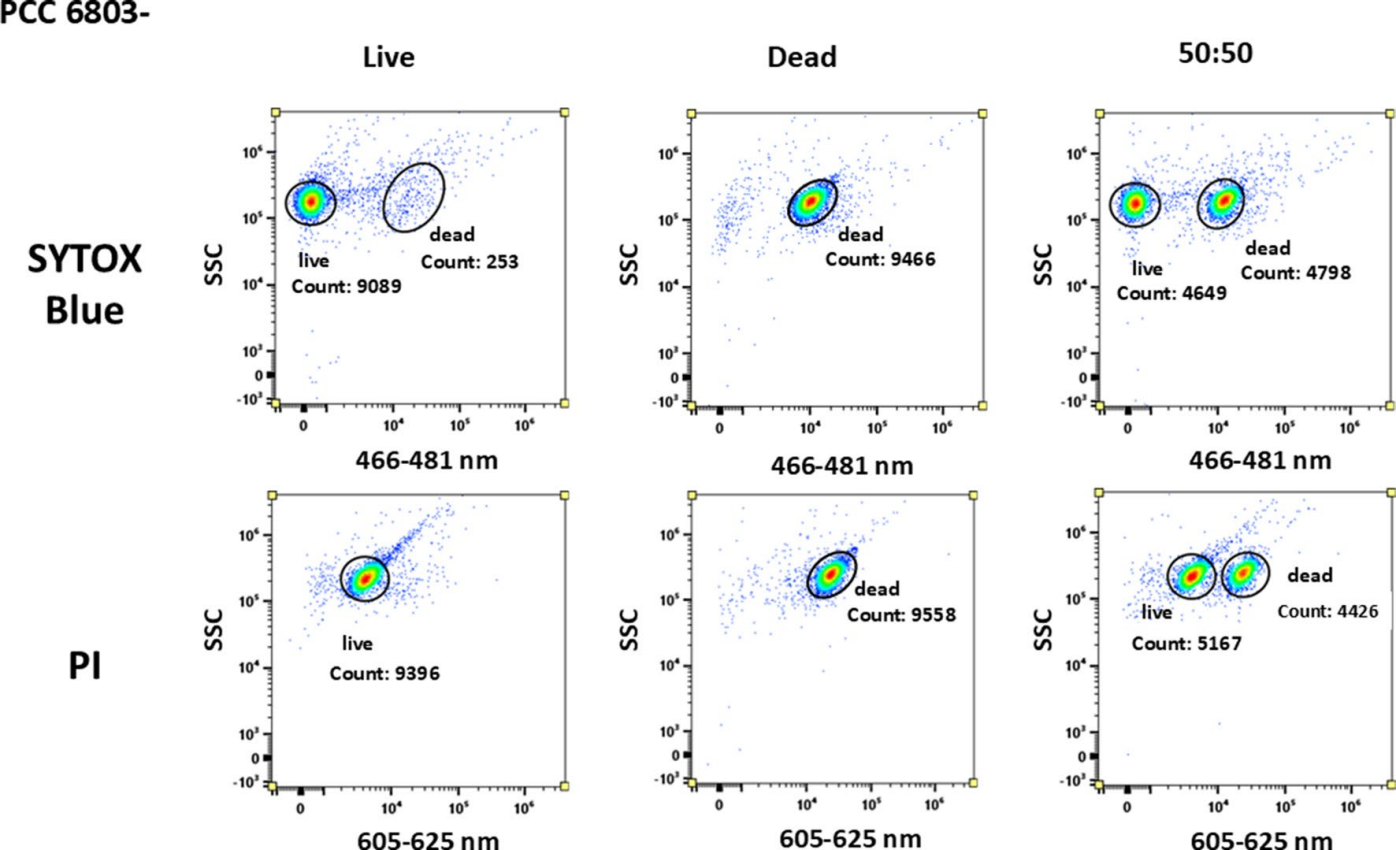
PCC6803 − signal in dot plots plot for SYTOX Blue and PI for all 3 types of samples. PI distinguishes between live and dead cells, but the signal of dead and live cells does not have that substantial difference in detected signal intensity. Live cells should possess fluorescent signal equal or close to 0, in the channel sensing wavelengths 605–625 nm. SYTOX Blue probe clearly distinguished live and dead cells, even in mixed (50:50) sample. The signal of the dead cells appears on the scale at the signal intensity of 10^4^ a. u. Axis ‘*y*’ exhibits the signal from SSC (side scatter); axis ‘*x*’ exhibits the range of emission wavelengths detected, 466–481 nm for SYTOX and 605–625 nm for PI

Figure 2 shows the signals of the tested probes on PCC6803 − (21 days long cultivation, no content of PHA). The PI distinguished the viable and the dead cells; however, ‘live’ cells have a signal intensity of 10^2^–10^3^ a.u., and the signal of dead cells is around 10^4^ a.u. in channel B6 sensing emission of 605–625 nm (Fig. 2). Nevertheless, distinguishing live and dead cells in mixed samples was still possible, but the signal of both populations is closer than in the case with the SYTOX probe.

The SYTOX probe provides the signal of dead cells around 10^4^ a.u. (Fig. 2), 0–10^2^ a.u. is the fluorescent signal of viable cells which correlates with demonstrated data from the manufacturer (demonstrated data in the protocol attached to the probe). The viable population is represented by a single population with almost 0 fluorescent intensity signal in channel V4 sensing emission of 466–481 nm, as the PCC6803 − is a ‘young’ culture, small number of dead cells are detected in the non-stressed culture. The signal of the dead cells appeared on the scale at the signal intensity of 10^4^. In the third sample (ratio of 1:1), two separate similar-size populations were detected in a mixed sample of ‘live’-dead cells which indicates that the SYTOX demonstrated good efficiency in distinguishing death cells in this cyanobacterial culture.

**Fig. 3:**
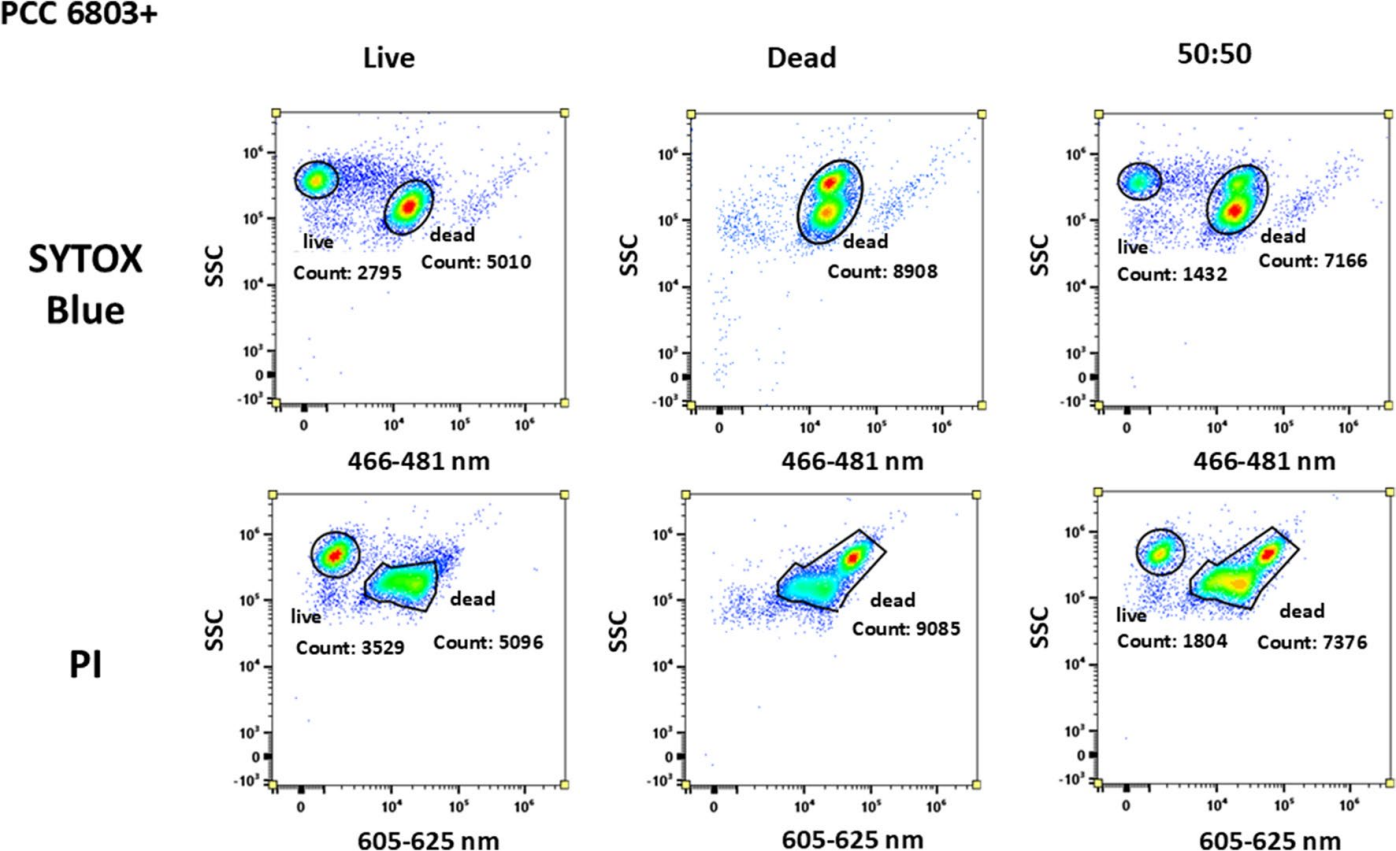
PCC6803 + signal in dot plots SYTOX Blue and PI for all 3 types of samples. PI distinguishes between live and dead cells, but the signal of dead and live cells does not have that substantial difference in detected signal intensity. Fluorescent signal of viable cells was shifted right closer to the signal of dead cells around 10^4^ a.u. SYTOX probe clearly distinguished live and dead cells, even in mixed sample. Fluorescent signal of live cells in channel 466–481 nm equals 0 a.u. Axis ‘*y*’ exhibits the signal from SSC (side scatter); axis ‘*x*’ exhibits the range of emission wavelengths detected, 466–481 nm for SYTOX and 605–625 nm for PI

Figure 3 shows the results of PCC6803 + (42 days long cultivation, higher content of PHA and pigments in cells). There are naturally occurring dead cells in the population of PCC6803 + culture (Fig. 3), as this culture is 42 days (stationary phase) old compared to PCC6803 − , which is 21 days old and thus in the middle exponential growing phase and containing minimum of dead cells (Fig. 2). In case of the PI, we observed the shift of the signal for viable as well as for dead cells. The signal for live cells was shifted left closer to 0, and the signal for dead cells was shifted right to higher intensities, but a kind of semi-population occurred in between. In the case of SYTOX, the differentiation of dead and viable cells was successful. We could identify the dead cells in the original sample because this culture is twice as old as PCC6803 − ; so, naturally, the dead cells occur in the population, and their presence is also supported by the senescence caused by nitrogen limitation. That is also the reason why in the case of the ‘50:50’ sample, the number of cells and the signal are higher for dead cells—because the original population already contained the dead cells. Two separate signals are also visible in histograms of the SYTOX channel V4 (left column).

Figures 8 and 9  in the Appendix confirmed these observations also for the second cyanobacterial culture CCALA192 − (no content of PHA, possible strong influence of autofluorescence) and CCALA192 + (42 days long cultivation, higher content of PHA and pigments in cells). The SYTOX Blue and the PI showed a similar signal pattern which means that both probes were capable of distinguishing between live and dead cells in the sample. PI again provided a fluorescent signal in the ‘live’ sample but despite that could differentiate mixed sample. The fluorescent signal of viable cells in the PI channel is suggesting that there could be slight interference in this channel as viable cells should be capable of excluding the dye and thus do not possess any signal. These results demonstrate that SYTOX Blue provides a trustful signal also for *Synechocystis salina* CCALA192 − .

Fluorescent probes were also tested on CCALA192 + (high content of PHA and comparable content of pigments with sample CCALA192 −); the data output is shown in Fig. 9 (in the Appendix). PI staining was in the case of CCALA192 + more confident, but the question is if the probe is sensitive enough. The SYTOX Blue could convincingly stain dead cells, but problems appeared in the original sample; the population of live cells provided a relatively wide distribution of intensity of fluorescent signal, which somewhat complicates its clear separation from the dead cells.

Based on our results, we established the staining protocol with SYTOX Blue for cyanobacteria in Table 3. This working protocol can be used both for cells of cyanobacteria containing PHA or with no PHA content, as well as, with different contents of autofluorescent pigments in cells.

**Table 3:** Staining protocol based on our findings during our work with cyanobacteria

Procedure	Specifications
Spin cell	Max 3000 g
Dilution	Approx. 10^6^ cells per mL (10^5^–5 × 10^7^)
Stain	1 μL, 10 min (max. 30 min) in darkness, RT, final concentration of the probe: 1 µmol/L
Measure	Channel V4 (466–481 nm) and SSC

#### Freezing–thawing challenge

The developed robust protocol for the determination of the viability of cyanobacterial cultures was employed for evaluation of the stress robustness of two *Synechocystis* strains with various PHA content in biomass (Fig. 10 in the Appendix). All the cultures were exposed to three repeating freeze–thaw cycles as freezing is an abiotic stress occurring frequently in various niches.

**Fig. 4:**
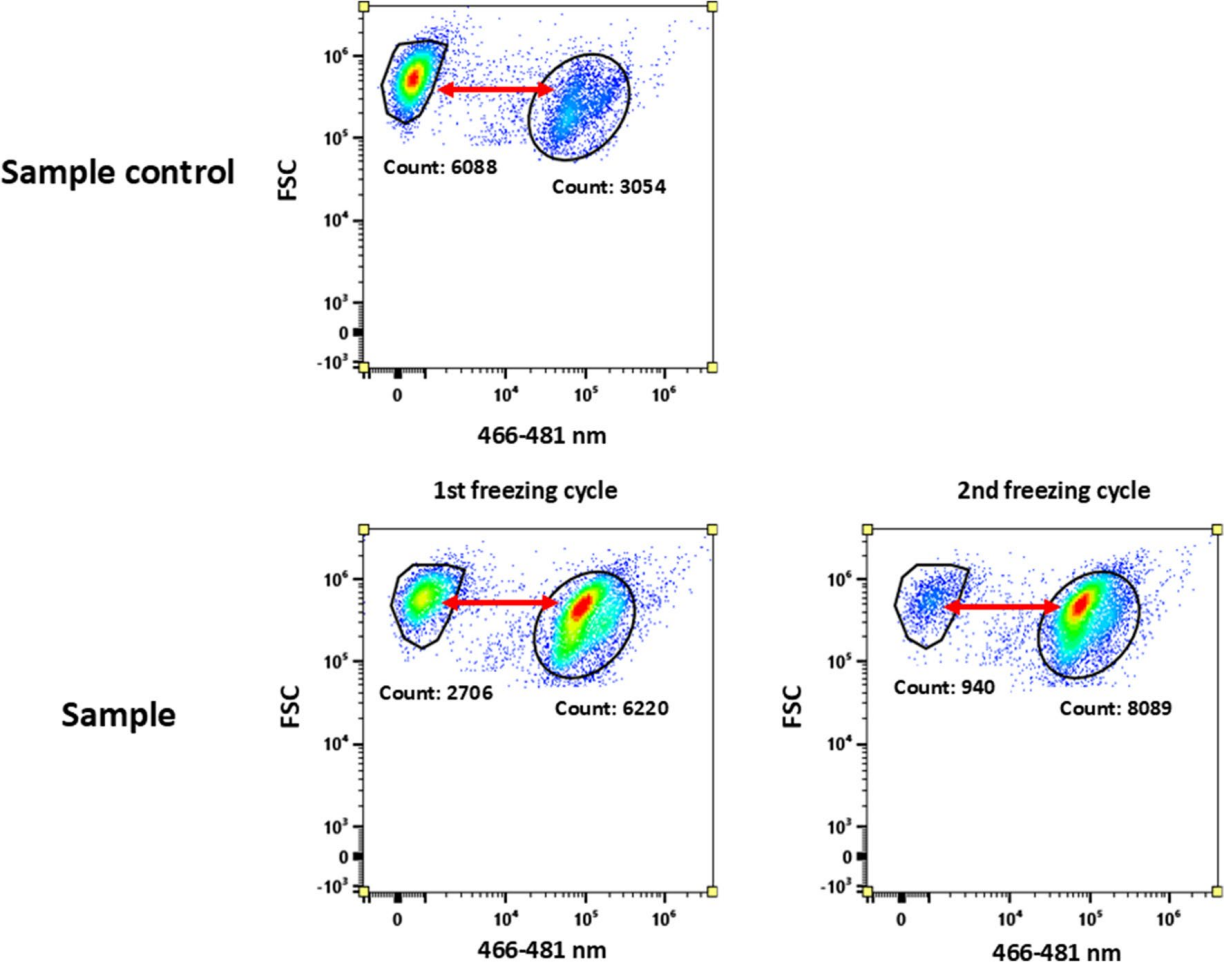
Probe SYTOX Blue was used to measure the viability of cells PCC6803 − exposed to freezing–thawing cycles. Sample control also represents the status of the culture before applying the stress conditions—which is represented by the ratio of viable and dead cells in the population. Usage of the SYTOX Blue on samples provided reliable results. We could observe decrease in number of viable cells and increase of the dead cells in the sample after 2nd freezing–thawing cycle

Three controls were applied in the experiment—two for the probe, one for the sample, and two testing samples (Fig. 4). Negative control represented by dead cells without the SYTOX Blue provided a signal with fluorescent intensity from 0 to 10^2^ a.u. in channel 466–481 nm (V4). Positive control represented by dead cells with SYTOX Blue provided a signal with fluorescent intensity of 10^4^ a.u. Sample control represents the status of the culture before applying the stress conditions. Dead cells are already appearing in the ratio of 2:1 viable cells to non-viable/dead cells.

The demonstration of cytograms for the freezing–thawing stress experiment is provided in Fig. 4. After the first freezing cycle two distinguished populations appear in the sample, the first population—the smaller one on the left—represents viable cells, and the second population—the bigger one on the right—represents dead cells. After the second freezing cycle, the number of cells dramatically decreased, and we could observe the raised number of dead cells. In other words, SYTOX Blue was reliable in recognising viable cells in the sample after exposure to freezing–thawing challenge.

Sample control was done for every sample undergoing the stress conditions. An appropriate number of cells (10,000) was used to calculate the number of viable cells during stress experiments. The viability of the cultures before the test is shown in Table 4.

**Table 4:** Viability of the cultures used for freezing–thawing test (It seems that increased viability could be potentially related to increase in PHA content in the cells.)

Sample	PHA (%)	Viability of the control (%) of viable cells	Number of live cells (count)	Number of dead cells (count)
6803 −	0.22	95.36 ± 0.07	9483	461
6803 +	5.76	43.94 ± 0.61	4270	5446
192 −	0.97	92.84 ± 0.07	9116	702
192 +	10.0	60.42 ± 1.45	5878	3850

**Fig. 5:**
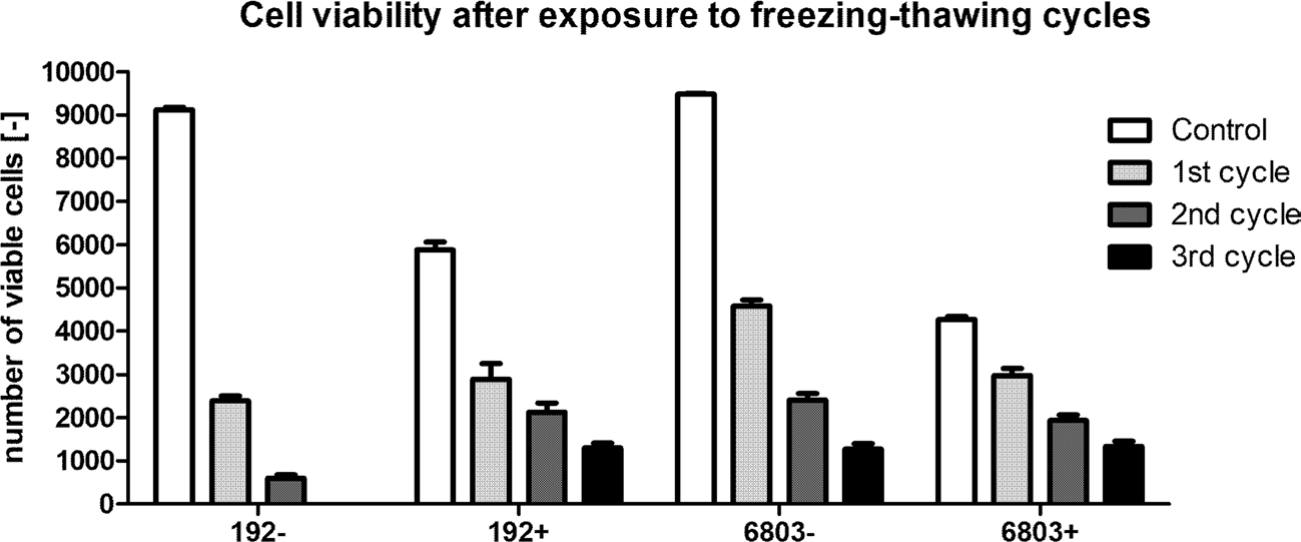
Viability of PCC 6803 and CCALA 192 after exposure to three freezing–thawing cycles in relation to PHA. Higher number of dead cells in the older populations (‘ + ’) is natural as the cultures were in the stationary phase of cultivation. Despite the lower number of viable cells in the older culture of CCALA 192, cell survival was higher. The error bar represents the standard deviation

CCALA192 + as well as PCC6803 + showed substantially higher viability in comparison to cultures with low PHA content (Fig. 5). The results of sample controls as well as tested samples are demonstrated as an average of three biological replicates.

### Electron microscopy

Electron microscopy was applied to visualise cyanobacterial cells to visually prove the presence of PHA granules and to visualise cell size and other cell components to compare CCALA 192 to model PCC 6803. For these purposes, we analysed cyanobacterial cells and their intracellular space by transmission electron microscopy (TEM) and cryogenic scanning electron microscopy (cryo-SEM).

#### Visualisation of cyanobacteria cells by TEM

**Fig. 6:**
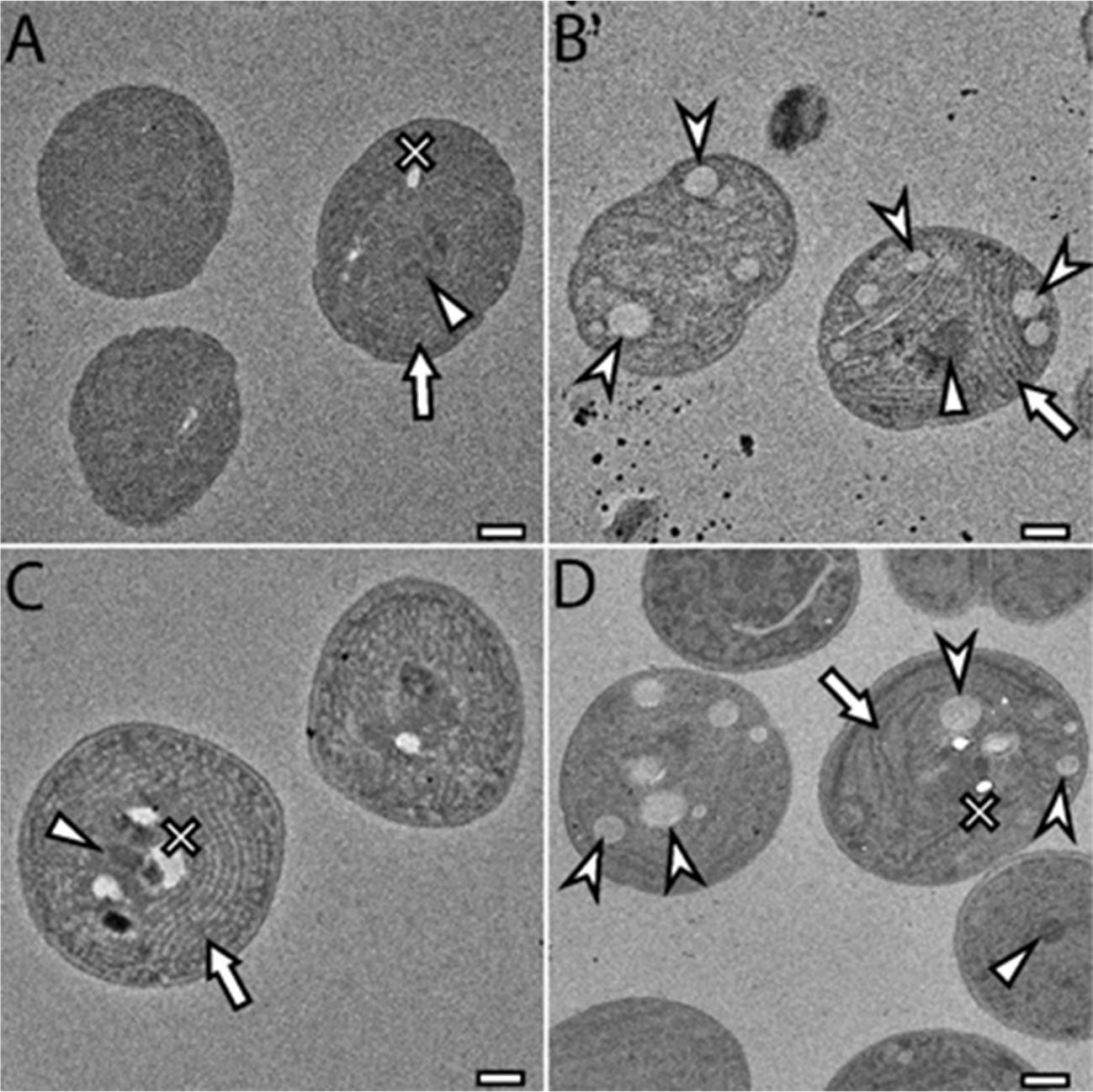
Intracellular structures of cyanobacterial cells imaged by TEM. **A** 192 − ; **B** 192 + ; **C** 6803 − ; **D** 6803 + . Intracellular structures marked by an arrow: thylakoid membranes; arrowhead: PHA granules; triangle: carboxysome; cross: washed-out granule. A ‘washed-out granule’ is a hole left in the ultrathin section of imaged cells. This artefact is commonly present when imaging cells with complex cell wall, that are difficult to infiltrate even with special resins. Due to the poorer infiltration (compared to different microbial samples) and complex intracellular ultrastructure, some granules in the cell can be washed out during the sample preparation procedure or fall out during the sample sectioning, leaving a hole in the sample, whose origin cannot be clearly identified. Scale bar: 1 μm

Cyanobacteria from genus *Synechocystis* are strictly unicellular (Kaneko et al. 1996) and shape of the cell is round. Cell is surrounded by an outer and cytoplasmic membrane. Inner space is occupied mainly by thylakoid membranes (Van de Meene et al. 2006). In the cyanobacterial cell, several structures can be observed (Fig. 6) including carboxysomes, PHA granules, or thylakoid membranes. The amount of PHA measured by gas chromatography showed higher amounts in 192 + and 6803 + , which was also confirmed by TEM (Fig. 6) that showed more PHA granules (marked by arrowhead) in 192 + and 6803 + than in 192 − and 6803 − . In Fig. 6, the presence of carboxysomes (marked by a triangle), PHA granules, and thylakoid membranes (marked by an arrow) was proved and correlates with previous studies (Van de Meene et al. 2006). All cell components are easily distinguishable.

#### Visualisation of cyanobacteria cells by Cryo-SEM

Apart from TEM, the Cryo-SEM technique was used to complete the visualisation of the cells. The same micro-compartments were observed in the microscope as visible in Fig. 11 in the Appendix. There are visible PHA granules in the populations of PCC6803 + (D) and CCALA192 + (B). More granules are likely to be observed in other layers of the sample and can occur in another fracture plane (cut) of the cell. Unfortunately, some samples showed a high charging effect that could not be removed and is thus observable in the images.

## Discussion

### PHA content

It has been reported previously that PHA does not play a role just as a storage compound for carbon and energy but also helps heterotrophic bacteria to survive stress conditions such as freezing, osmotic pressure, or UV radiation (Obruca et al. 2020; Pernicova et al. 2020). Accordingly, we would like to investigate the role of PHA presence in the cyanobacterial cells and the consequences of the proper stress dosses to positively influence the production of PHA and at the same time contribute to the viability of the cells themselves. However, as already described above, cyanobacteria belong to much more complicated microorganisms than heterotrophic bacteria due to the presence of membranes, intracellular pigments, etc. Based on cell complexity in cyanobacteria, it was not possible to use established protocols for determining the viability of PHA-containing cells that were exposed to a certain stress at a defined dose via flow cytometry, mainly because of the autofluorescence of pigments. Therefore, firstly, we had to implement a reliable method with a suitable viability probe usable for cyanobacteria cells. For this purpose, we selected two cyanobacterial strains, model cyanobacterium with respect to PHA metabolism *Synechocystis* PCC 6803 and its taxonomical relative *Synechocystis* CCALA 192. Despite their relatedness, they vary in several aspects, for example, CCALA 192 tolerates higher salinity, prefers lower cultivation temperature, and naturally produces more PHA than PCC 6803 (Meixner et al. 2022a).

### Pigment content

**Fig. 7:**
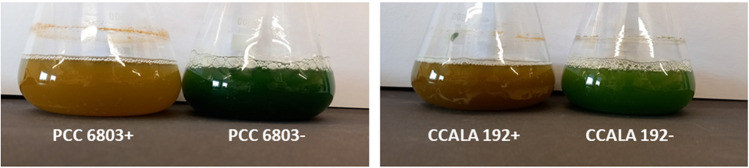
Cultures of different age and PHA content, visible colour change due to chlorosis. Older yellowish cultures are rich in PHA (marked as ‘ + ’); younger green cultures have low PHA content (marked as ‘ − ’)

To achieve two different amounts of PHA in one culture, we used different cultivation times for the cells, which differed not only in PHA content but also in pigment content. However, the pigment content, mainly the amount of chlorophyll and carotenoids, does not indicate only age but also the condition of the culture. Thus, the change of colour is a very important indicator originating from the degradation of chlorophyll and phycocyanin caused by nitrogen limitation; this colour-change is called chlorosis (Spät et al. 2018) (Fig. 7 in the Appendix). Similarly in plants, N-deficiency induces senescence, i.e. reduction of the content and/or the activity of photosynthetic enzymes, and causes the degradation of the photosynthetic enzymes (namely, RuBisCo, phosphoenolpyruvate carboxylase, and pyruvate orthophosphate dikinase) (Mu and Chen 2021).

### Identification of suitable fluorescent probe

#### Testing chosen fluorescent probes

As both chosen cyanobacterial strains are capable of PHA synthesis, research aimed to measure the viability after stress exposure to investigate acute stress and prolonged stress in case of hormesis. For this purpose, we chose three viability probes considering the complexity of cyanobacterial cells, concretely PI, and SYTOX Blue to establish a reliable method for flow cytometry. Firstly, we used the propidium iodide (PI), which is commonly used for cell viability assessment. The last tested probe SYTOX Blue was chosen based on its emission spectra and also by the probe’s suitability for flow cytometry. Both, PI and SYTOX Blue, belong to the membrane-impermeable fluorescent dyes. PI was used to investigate the effect of paraquat and copper on marine and freshwater microalgae, with the length of exposure of the number of cells with damaged membranes increased (Franqueira et al. 2000). Limits of PI were investigated while using the PI as a non-viable cell indicator in filamentous cyanobacteria, when PI did not work properly (Johnson et al. 2015) and SYTOX Blue can be used as an alternative to PI. Unfortunately, SYTOX Blue stains viable filamentous cyanobacterial cells similarly as PI (Johnson et al. 2016). This phenomenon may be possibly caused by the existence of intracellular channels that serve as a corridor for nutrient passage between cells in the filaments (Mullineaux et al. 2008). SYTOX Blue belongs to the family of SYTOX probes along with the widely used SYTOX Green, probes from this SYTOX family bind to DNA of cells with breached membranes (Krause et al. 2007; Thakur et al. 2015). SYTOX Green is widely used for cell membrane integrity studies employing flow cytometry with excitation wavelength 504 nm and emission wavelength 523 nm. In case of cyanobacteria, SYTOX Green was used for monitoring the influence of experimentally generated turbulence of *Microcystis aeruginosa* (Regel et al. 2004), the effect of chlorination (Daly et al. 2007), and the cell membrane integrity of *Microcystis aeruginosa* during exposure to hydrogen peroxide (Mikula et al. 2012), evaluating the impact of various chemicals on cyanobacterial cell integrity (Fan et al. 2013) and to evaluate *Synechococcus* sp. PCC7002 growth on the gravity belt filtrate (Korosh et al. 2018). The cell integrity is related to viability of the cell, as cells without intact membranes are not capable of metabolic activity, their internal structures are exposed to the environment and thus can be classified as dead (Nebe-von-Caron et al. 2000). SYTOX Blue has excitation wavelength of 444 nm, and emission maximum is around 480 nm. In comparison to PI and SYTOX Green, the emission wavelength is shorter; this advantage minimises the potential interference with cyanobacteria autofluorescence. As mentioned previously, SYTOX Blue is being used for staining non-viable cells; SYTOX Blue was used for labelling single bacterial cells (Krause et al. 2007), to visualise non-viable cells of aerobic granules from activated sludge (Adav and Lee 2008) or to identify non-viable cells in plant tissue using confocal microscopy (Truernit and Haseloff 2008). Johnson et. al. demonstrated that SYTOX Blue does not interfere with green fluorescence and at the same time does not add any additional blue fluorescence (Johnson et al. 2016). Autofluorescence differs depending on cyanobacteria strain and cultivation conditions. Chlorophyll exhibits autofluorescence in the red region and absorbs blue and red light. Unlike PI, SYTOX Green does not interfere with chlorophyll, with PI does interfere with chlorophyll minimally (Persichetti et al. 2020; Croce 2021). In the case of *Synechocystis* CALU 1336, autofluorescence of chlorophyll was detected in the red region (two maxima: 656 and 682 nm) while excited by a 488-nm laser (Rumyantsev et al. 2017). If phycobilins were present in our cyanobacteria, they could interfere with SYTOX Blue, but at an excitation of 400 nm, this is not relevant because the excitation maxima are as follows: phycocyanin 656–678 nm, phycoerythrin 500–550 nm, phycocyanobilin 450–490 nm, and again, it depends on the microorganism and cultivation conditions. As mentioned previously in chapter with pigments, cyanobacteria have both pigments—phycocyanin and chlorophyll, both can be excited by the blue laser (488 nm), but the chlorophyll excites better with the blue lasers. Due to that phycocyanine could possibly interfere with PI. Autofluorescence was not so intense as to obscure our probes according to controls. Secondly, due to the use of lasers and a spectral cytometer with very narrow bands, we also mitigated the degree of interference. So, usage of SYTOX Blue should not be affected by autofluorescent signals.

#### Viability of PHA-poor cultures

For comparison of selected viability probes for cyanobacterial cells, we tested three probes on young pigmented cyanobacterial cells without any PHA to obtain relevant data, concretely PCC6803 − (see Fig. 2) (Trautner and Vermaas 2013). The usage of PI led to the recognition of viable and dead cells in mixed samples. Nevertheless, the signal should be provided only by dead cells when the PI binds to DNA.

**Fig. 8:**
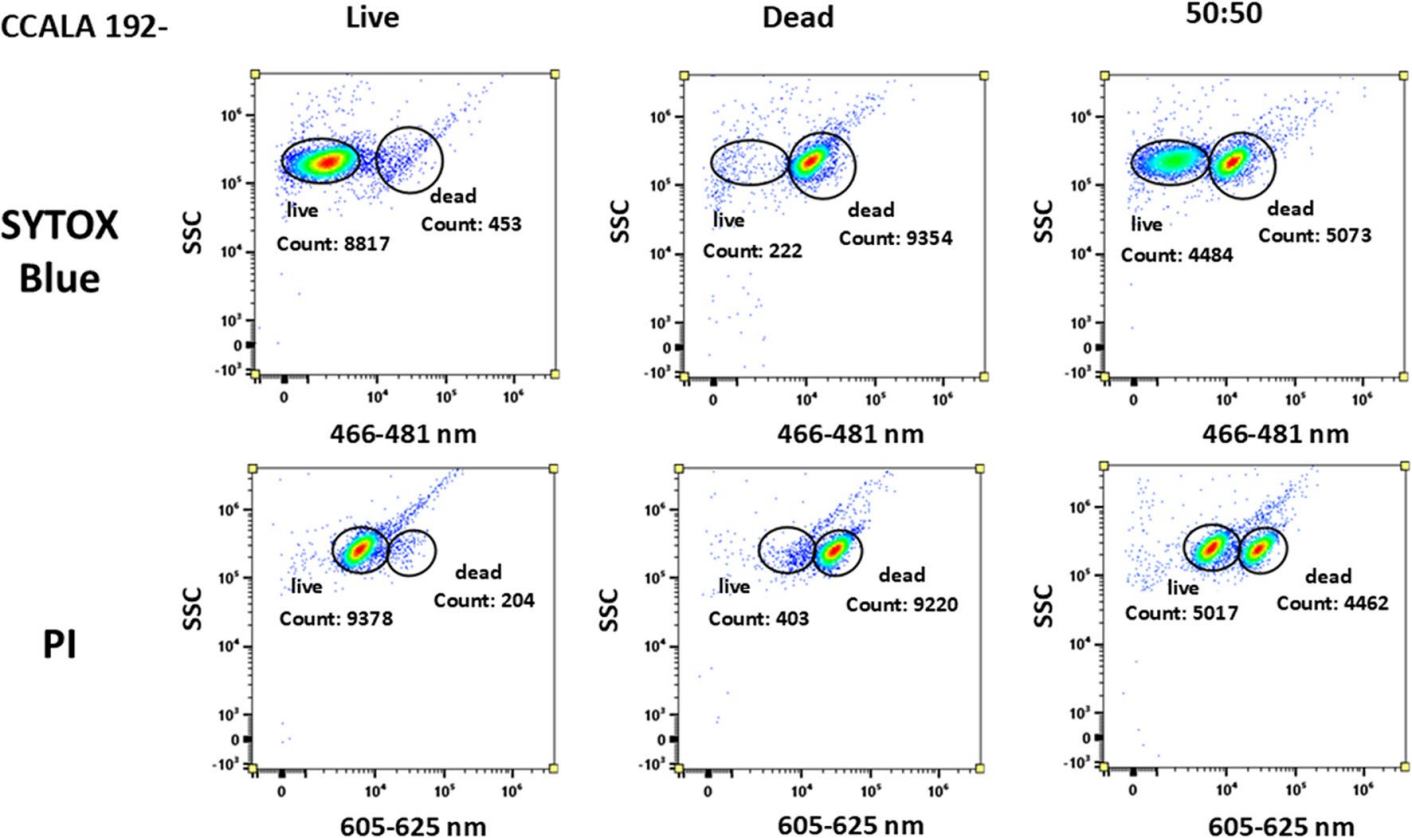
CCALA 192 − dot plot for SYTOX Blue and PI for all 3 types of samples. SYTOX Blue probe clearly distinguished live and dead cells, even in mixed sample; PI and SYTOX Blue distinguished live and dead cells but the signal of dead and live cells does not have that big difference in detected signal intensity. Limitation of PI probe could be live cells possess signal on the collecting channel for PI probe. In case of live cells in the channel for SYTOX Blue emitted, signal is lower and close to 0. Axis ‘*y*’ exhibits the signal from SSC (side scatter); axis ‘*x*’ exhibits the signal of the probe in channel for SYTOX Blue and PI

**Fig. 9:**
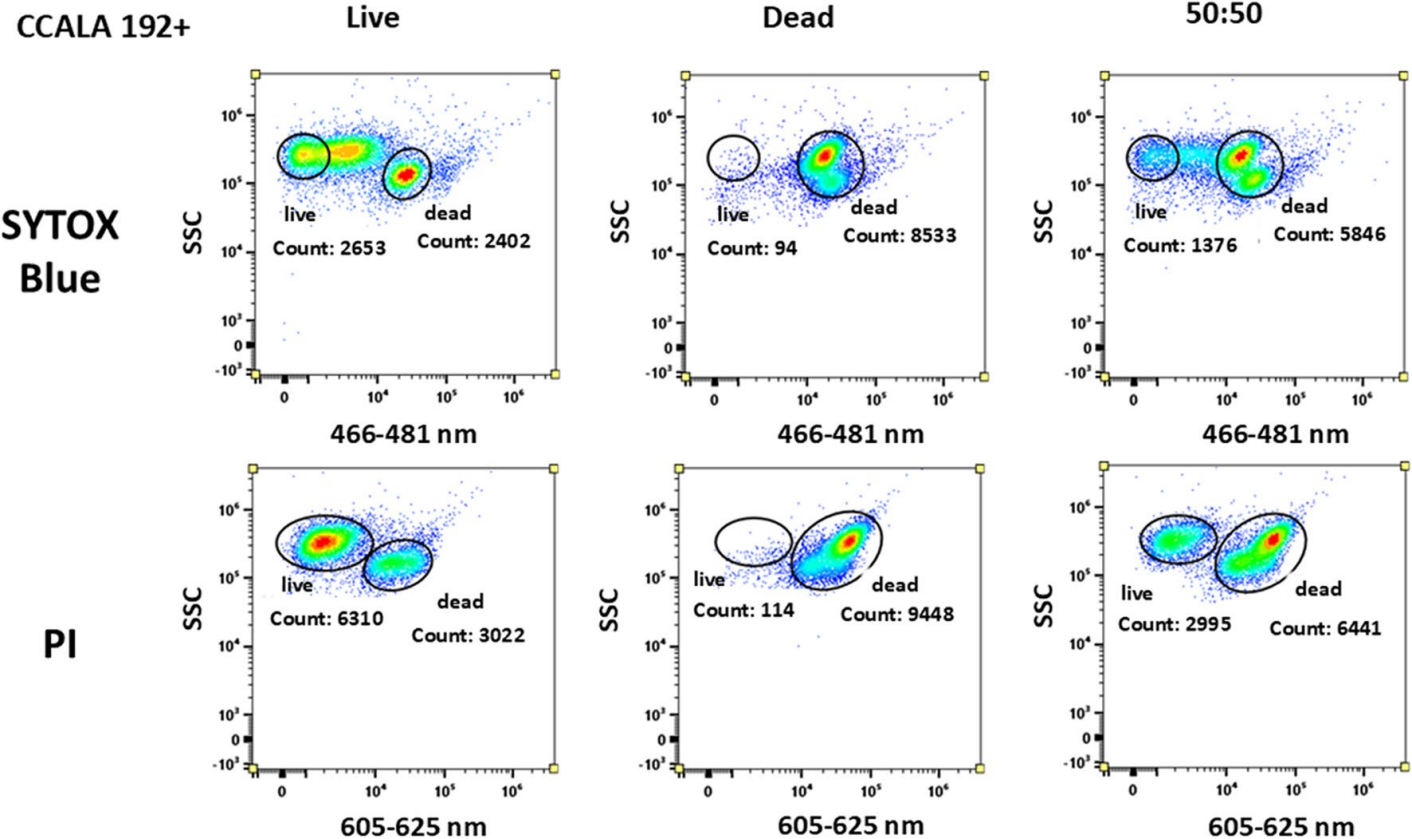
CCALA 192 + dot plot for SYTOX Blue and PI for all 3 types of samples. SYTOX Blue distinguished live and dead cells but the signal of dead and live cells is not separated; signal of live cells is shifted; PI probe clearly distinguished live and dead cells, even in mixed sample. Axis ‘*y*’ exhibits the signal from SSC (side scatter); axis ‘*x*’ exhibits the signal of the probe in channel for SYTOX Blue and PI

The question could be what provides the signal with the intensity of 10^3^ a.u. in the original sample when the signal should be provided only by cells with compromised membranes? Most probably, the signal is provided by some of the cyanobacterial photosynthetic pigments. As the population is older and cell chlorosis occurs, more metabolites and pigments are produced, and the autofluorescence of some of them could interfere with the fluorescence of the probe. However, for the determination of the overall/relative viability of the culture, the probe is still sufficient. Moreover, the last viability probe SYTOX Blue proved the best and clear discernment between dead and live populations in a mixed sample in comparison to PI that have the same strategy of binding to nucleic acids in dead cells (Thakur et al. 2015). For further analysis of selection of the best and reproducible viability probe for cyanobacteria, we also performed the experiments on cyanobacterial strain CCALA192 − and CCALA192 + (see Figs. 8 and 9 in the Appendix) that demonstrated and proved the data that were obtained with strain PCC6803 − .

#### Viability of PHA rich cultures

In addition to determining the viability of green/young cyanobacterial cells, we also tested a suitable viability probe for cyanobacterial cultures that had undergone chlorosis, i.e. higher concentration of carotenoids (Fig. 1) and the appearance of intracellular secondary metabolites. Considering the comparison of various cell complexity of different aged cyanobacterial cultures, we verified the success of the viability probe SYTOX Blue also on PHA accumulating culture CCALA192 + and PCC6803 + . From the data (Figs. 3 and 9), we observed that the wide distribution of fluorescent signal intensity was spread from 0 to 10^3^ a.u. in channel V4 (466–481 nm), and a new peak appeared between the peaks for viable and dead cells in a ‘live’ sample. This could be the population of senescent cells or cells with a higher amount of PHA, which could reflect/scatter the light (Slaninova et al. 2018) and cause the false signal of the probe.

The fluorescence of the SYTOX Blue probe also most probably partially interferes with the autofluorescence of carotenoids in chlorotic cells of CCALA192 + . Carotenoids represent a large group of biological chromophores; the majority of carotenoids exhibit absorption in the visible region of the spectrum in the range between 400 and 550 nm, resulting in their yellow-orange colour. The excitation wavelength of β-carotene is between 480–520 nm, which is similar to SYTOX Blue (Gillbro and Cogdell 1989). Nevertheless, from our obtained data (Fig. 1), the content of carotenoids in CCALA192 + was twice the amount found in PCC6803 + (up to 20 ug/mL) and four times higher than in young green cultures of both strains (Fig. 1). This non-isolated mixture of carotenoids and synthesis intermediates can interact in the energy transfer and therefore change the absorption maxima. Furthermore, we hypothesise that non-isolated carotenoids may be associated by hydrophobic interaction with membranes and proteins, causing a shift in absorption wavelengths. This could be the explanation why the probe works for PCC6803 + in the stage of chlorosis (orange-yellow colour of the culture) and does not work that precisely for CCALA192 + .

Except for fluorescent probes, autofluorescent signals may be useful for fluorescence-based detection and monitoring in water quality assessment studies (Takahashi 2019). In the study from 2023, fluorescence microscopy and spectroscopy techniques were used to directly observe and analyse the natural fluorescence emitted by the microorganisms when excited by specific wavelengths of light. The autofluorescence signal can reflect the abundance and distribution of photosynthetic pigments like chlorophyll *a* and/or phycobiliproteins. Changes in the autofluorescence intensity of spectral profile may indicate variations in photosynthetic activity, cellular metabolism, or environmental stressors affecting the organisms (Brentjens et al. 2023).

#### Freezing thawing stress challenge

We employed the viability determination protocol to assess the resilience of cyanobacterial cultures when subjected to repetitive freezing–thawing challenges. Freezing and thawing represent prevalent abiotic stressors that inflict numerous detrimental effects on prokaryotic cells. At subzero temperatures, ice crystals initially form in the extracellular medium surrounding the cells, inducing an effective osmotic stress as the solute concentration in the vicinity of the cells is excluded into a diminishing volume of solvent. The second significant deleterious event observed during cell freezing is the proliferation of intracellular ice crystals. The precise mechanisms of damage resulting from intracellular ice crystals remain elusive, but they probably encompass the physical destruction of membranes, the formation of gas bubbles, and the disruption of organelles (Fuller 2004). Due to their more complex intracellular structure, cyanobacterial cells are more prone to the negative effect of freezing than other heterotrophic bacteria.

PHA emerges as a crucial metabolite in conferring stress robustness to bacterial cells exposed to the challenges of freezing–thawing cycles. For instance, in *Cupriavidus necator*, a model strain for PHA metabolism, the presence of PHA granules significantly enhanced the survival of bacterial cultures subjected to freezing–thawing stress. This improved resilience is likely attributed to the elevated intracellular concentrations of 3-hydroxybutyrate, a potent cryoprotectant and a PHA monomer. Additionally, the distinctive mechanical properties of PHA granules contribute to the stress-shielding effect of PHA (Slaninova et al. 2018; Obruca et al. 2020). The significance of PHA (Novackova et al. 2022) at subzero temperatures is further indicated by the observation that many cold-environment-adapted microorganisms demonstrate the capacity for PHA synthesis (Ciesielski et al. 2014; Goh and Tan 2012; Kaartokallio et al. 2013). Based on our results, we hypothesise that PHA could possibly have also a pivotal role in the survival of cyanobacteria during freezing–thawing challenges. Cyanobacterial phenotypes rich in PHA (CCALA192 + , PCC6803 +) exhibit substantially higher survival rates when subjected to repeated freezing and thawing compared to their PHA-poor counterparts (CCALA192 − , PCC6803 −) (Fig. 5). This phenomenon of higher survivability of cyanobacterial PHA rich cultures is similar to the phenomenon observed in the case of heterotrophic bacteria. The important role of PHA for bacterial survival under hyperosmotic conditions was explored using *C. necator* as non-halophilic bacteria. The presence of PHA granules in cytoplasm protected *C. necator* cells against the harmful impact of hypertonic environments (Obruca et al. 2017). Same PHA-rich heterotrophic bacteria, *C. necator*, exhibited a higher survival rate while exposure to freezing compared to PHA-deficient cells (Obruca et al. 2016). These similarities led us to hypothesise if a higher number of viable cells is somehow connected to PHA content. Basically, if the role of PHA in cyanobacteria could possibly be the same as in heterotrophic bacteria. To definitely prove this hypothesis, more experiments should be done, e.g. analyse freezing–thawing survival rate in mutants unable of PHA synthesis.

While recognising the complexity of differences between the two phenotypes, it is evident that conditions promoting enhanced PHA accumulation generally elevate the freezing–thawing survival of cyanobacteria. Although these differences cannot be solely attributed to varying PHA contents, our results imply that conditions favouring increased PHA accumulation generally enhance the freezing–thawing survival of cyanobacteria. At the same time, other factors can contribute to survivability of the cyanobacterial cells including glycogen, osmotic molecules, or other unknown processes.

Presented protocol could be possibly used for viability confirmation in studies of nitrogen chlorosis of wild type *Synechocystis* cells (Forchhammer and Schwarz 2018) as well as for mutant cells (Spät et al. 2018). Viability of the cells exposed to nitrogen limitation, long-term starved cells, cells after nitrogen resuscitation could be easily measured during various growth phases, as well as during various time sloths. Similarly, the viability could be observed during experiments dedicated to investigation of carbon/nitrogen homeostasis (Forchhammer and Selim 2019), studies keen on salt stress response (Klähn et al. 2021), high-light induced stress (Kojima et al. 2022), and cold or another stresses (Mironov et al. 2019).

### Electron microscopy

As the cyanobacterial cells are more complex than other prokaryotic cells, subcellular micro-compartments such as carboxysomes (CBX) appear in the cells (Rae et al. 2013). Together with CBX, a typical attribute of *Synechocystis* cell is the thylakoid membrane (Rast et al. 2019). The thylakoid membrane is a crucial component of the cyanobacterial cell as the photosynthesis and cell respiration take place there (Frain et al. 2016). Cyanobacteria are complex cells; several ‘micro-compartments’ were already observed in the pictures from TEM (Nienaber and Steinitz-Kannan 2018). The samples for TEM are prepared as ultrathin sections, so more granules can be in different layers of the cell as well as the size of the granules can be larger—the size in the picture depends on the layer; in other words, the size depends on where the cell was cut. The presence of PHA granules was proven by TEM (Fig. 6) and thanks to that we can suggest that the presence or absence of PHA granules in cyanobacterial cells of genus *Synechocystis* does not affect the application of SYTOX Blue fluorescent probe for investigation of cell viability via flow cytometry.

## Conclusion

The objective of this study was to develop a protocol for determining the cell viability of cyanobacterial cultures and to assess their survival under selected stress conditions. Among the various fluorescent probes tested, staining cells with SYTOX Blue demonstrated high reliability. We are fully aware that our technique has limitations as well as all of the viability assays. We state that both PI and SYTOX Blue can be used; nevertheless, SYTOX is slightly better in the stain index. It is known that SYTOX Blue does not work as a non-viable cell indicator in case of filamentous cyanobacteria due to intracellular transporting systems which led to non-specific binding of the probe. In case of single-cell cyanobacteria from the genus *Synechocystis*, SYTOX Blue showed reliable results as the cells are not connected by channels used for the cell communication or transportation of chemical substances. Viable cells exhibit either no or low fluorescent signals, minimising interference with the fluorescence of the probe. Notably, the presence of PHA along with pigments does not impede the application of SYTOX Blue for establishing cell viability in cyanobacterial cells. The developed viability assay was subsequently employed to evaluate the survival rate of cyanobacterial cultures subjected to repeated freezing–thawing, a common abiotic stressor. The results of this stress challenge led us to the hypothesis—despite the relatively low PHA content in cyanobacterial cultures compared to heterotrophic bacteria, PHA granules probably could play an important role in the survival of cyanobacterial cells when abruptly exposed to freezing and subsequent thawing. Nevertheless, to confirm the hypothesis, that PHA contributes to survivability of cyanobacterial cells when exposed to freezing–thawing challenge, more experiments should be done.

## Appendix

**Fig. 10:**
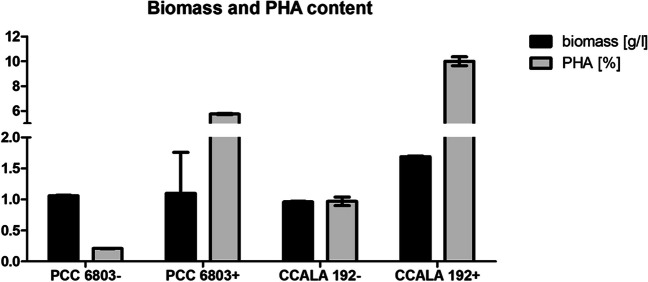
PHA amount in the cultures used for testing the SYTOX Blue on samples exposed to freezing–thawing cycles. The PHA content in older cultivation harvested in stationary phase (‘ + ’) ranged between 6 and 10% of PHA in cdw. The error bar represents the standard deviation

**Fig. 11:**
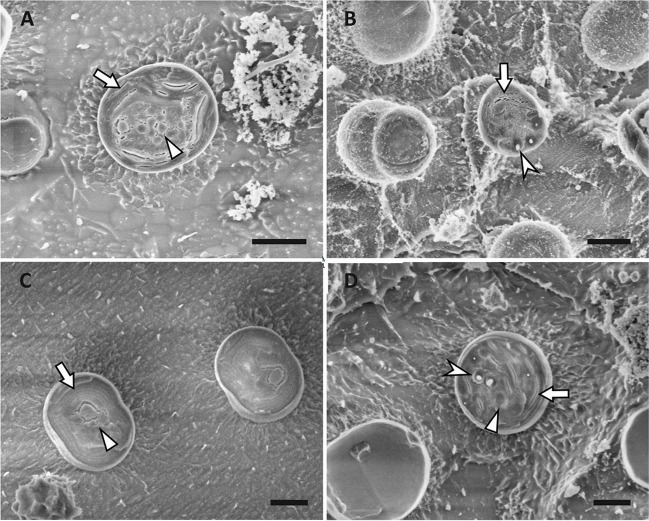
Intracellular structures of cyanobacterial cells imaged by cryo-SEM. **A** 192 − ; **B** 192 + ; **C** 6803 − ; **D** 6803 + . Intracellular structures marked by an arrow: thylakoid membranes; arrowhead: PHA granules; triangle: carboxysome. Scale bar: 1 μm

Figures 7, 8, 9, 10 and 11.

## Data Availability

I do not have any research data outside the submitted manuscript file.
